# Resolved Hyperfine at L-band for High-Spin CoEDTA, A Model for Co Sites in Proteins

**DOI:** 10.3390/ijms20102385

**Published:** 2019-05-14

**Authors:** William E. Antholine

**Affiliations:** Department of Biophysics, Medical College of Wisconsin, Milwaukee, WI 53226, USA; wantholi@mcw.edu; Tel.: +414-955-4032

**Keywords:** electron paramagnetic resonance, EPR, L-band, 1.37 GHz, high-spin CoEDTA, resolved Co hyperfine lines at ***g_eff_***-mid

## Abstract

Low-frequency electron paramagnetic resonance (EPR) spectra were obtained for the Co complex of ethylene diamine tetraacetic acid (CoEDTA). It was found that the cobalt hyperfine at ***g_eff_***-mid is better resolved at a low frequency, L-band (1.37 GHz), and not resolved at X-band (9.631 GHz), which is the conventional frequency used for most spectra for metal complexes. Resolved cobalt hyperfine lines lead to additional EPR parameters like ***A***-mid for cobalt and a more-accurate determination of ***g***-mid. Resolved hyperfine lines in the L-band, but not the S-band, spectra were obtained at a concentration of 1 mM. Knowing these additional EPR parameters provides a means to better determine the electron density in the ground state orbital for each cobalt complex, as well as to determine differences upon a change of ligation. If zinc sites can be replaced by cobalt, the cobalt spectra for these sites will enhance the characterization of the zinc sites.

## 1. Introduction

Hyperfine lines in electron paramagnetic resonance (EPR) spectra at low frequencies are better resolved because ***A***-strain and ***g***-strain are minimized and the line widths are narrower [1,2,3,4,5]. Copper hyperfine lines at low microwave frequencies, but not usually at X-band (the commercial frequency where most spectra are obtained), are so narrow in the ***g****_ll_* region that superhyperfine lines from the nitrogen donor atoms are resolved. The pattern for the nitrogen superhyperfine lines determines the number of nitrogen donor atoms for the cupric complex. For example, the site for Cu-serum albumin and Cu-pMMO has four nitrogen donor atoms, while the cupric binding site for the prion protein, Cu-prion protein, has three nitrogen donor atoms and one oxygen donor atom [5,6,7]. Use of the Froncisz–Hyde loop-gap resonator at low microwave frequencies makes it possible to use EPR tubes at X-band (4 mm outside diameter quartz tubes containing 0.3 mL of sample) at low frequencies instead of tubes containing about 30 mL of sample using a conventional resonator [2,3].

The hyperfine values for *g*-parallel for cupric complexes are usually between 130 Gauss (G) and 200 G [1]. The hyperfine values for cobalt (Co) complexes range from about 100 G to 10 G. It is shown in this paper that the hyperfine coupling for these Co complexes is large enough, for example 50 G, to be sensitive to strains including the *A*-strain, *g*-strain, and *D*-strain at L-band. The line width for Co hyperfine lines usually is not well enough resolved at S-band, (3.3 GHz) [8]. In this study, hyperfine lines for the Co complex of ethylene diamine tetraacetic acid (CoEDTA) are better resolved at L-band, but not at S-band or X-band. The L-band and S-band spectra for a high-spin Co-doped ceramic where the Co hyperfine is resolved at both frequencies was recently published [9]. Although the L-band spectra for several high-spin Co-sites were obtained previously [10,11,12], interpreting the spectra was difficult. Now, the similarity between some of the L-band spectra and the spectra for the Co-doped ceramic supports the interpretation of the L-band spectra, and the change in spectral shape for other Co-sites shows that the spectra are sensitive to EPR parameters not obtainable for spectra taken at the commercial frequency, X-band. Moreover, the simulation of the L-band spectrum is verified by experimental L-band spectra. EPR parameters from the acquisition of low-frequency L-band spectra will provide data that can be used to interpret the electron spin density in the molecular orbital for which the hyperfine values are assigned. Not only is this detail important for Co complexes, it also is important for zinc sites for which Co can replace zinc [8].

## 2. Results

### 2.1. EPR Spectra for CoEDTA at X-band (9.631 GHz) and S-band (3.3 GHz) Where Hyperfine for g_eff_-mid is not Resolved

**Figure 1:**
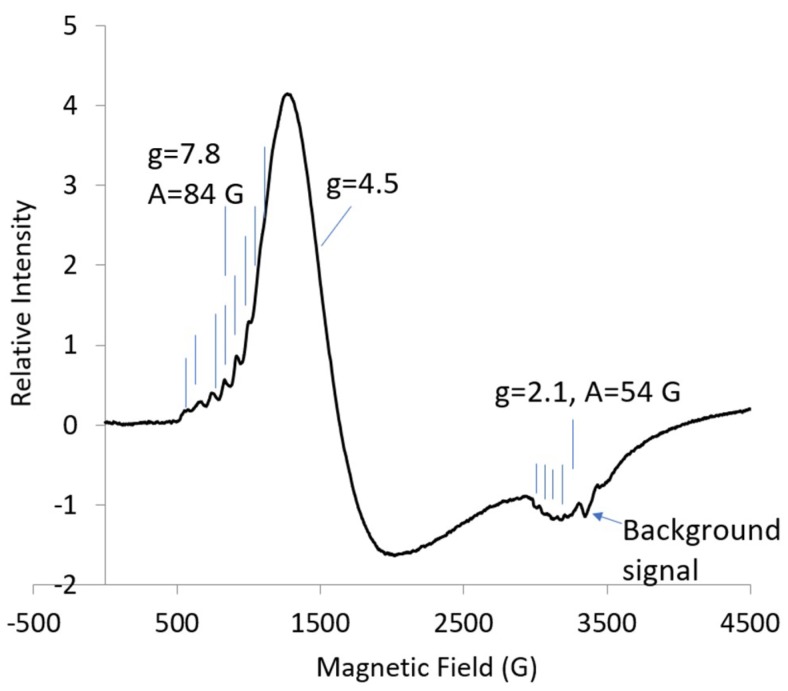
X-band (9.631 GHz) electron paramagnetic resonance (EPR) spectrum for the Co complex of ethylene diamine tetraacetic acid (CoEDTA) (1 mM): temp., 8 K; microwave power, 10 dB; nine scans averaged. Vertical lines indicate the Co hyperfine lines, eight for ***g*** = 7.8 and four of eight for ***g*** = 2.1, also indicate the g-values.

The EPR spectrum for the Co complex of ethylene diamine tetraacetic acid (CoEDTA) at 8 K is shown in Figure 1. Low-field hyperfine lines with ***g_eff_*** = 7.83 and ***A*** = 84 G were attributed to the | ± 3/2> state. At ***g*** = 2.14, hyperfine lines with ***A*** = 54 G were resolved and attributed to the [| ± 1/2> state. The rest of the spectrum for the | ± 1/2> state was not resolved (Figure 1). The eight lines about ***g_eff_*** = 7.8 decreased when the temperature decreased from 10 K to 5 K, while the change in the intensity of the lines attributed to ***g_eff_***-max and ***g_eff_***-mid at about ***g*** = 4.4 in Figure 1 remained about equal although the line shape changed (not shown). The | ± 1/2> state was the ground state. The shape of the lines for the | ± 1/2> state in Figure 1 indicated that the g-value of 2.14 was ***g_eff_***-min with ***g_eff_***-mid about 4.5.

**Figure 2:**
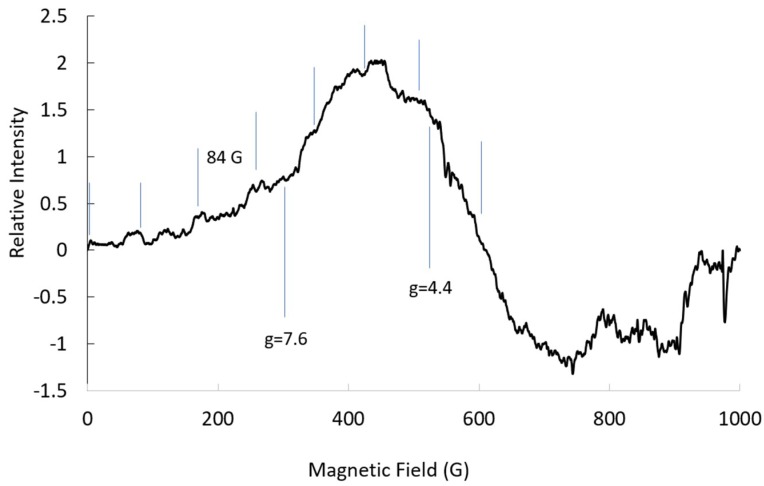
S-band (3.229 GHz) EPR spectrum for CoEDTA: temp., 18 K; microwave power, 16 dB; 9 scans averaged. Vertical lines indicate the Co hyperfine lines, eight (assuming the center is ***g*** = 7.6).

At S-band (3.3 GHz), seven of the Co hyperfine lines for | ± 3/2> were resolved, but the lines were barely detectable when compared to the intensity of the central line for | ± 1/2> (Figure 2). The width of the line for ***g***-mid suggests that the Co hyperfine was about 50 G and the ***g***-crossover was about four. It would be difficult to assign either hyperfine lines or ***g***-values without the parameters from the X-band spectrum. There was little resolution of the Co hyperfine line for ***g_eff_***-mid; the same was similar for other Co sites [8].

### 2.2. High-spin CoEDTA, Hyperfine Lines from **g_eff_**-mid Resolved at L-band (1.37 GHz)

**Figure 3:**
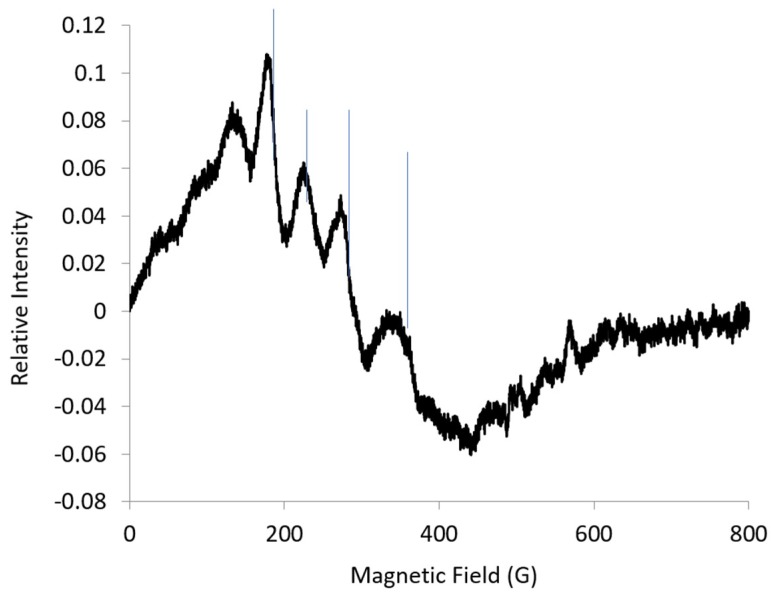
L-band (1.37 GHz) EPR spectrum for CoEDTA (1 mM): temp., 18 K; microwave power, 8 dB; 25 scans averaged. Vertical lines mark four hyperfine lines.

**Figure 4:**
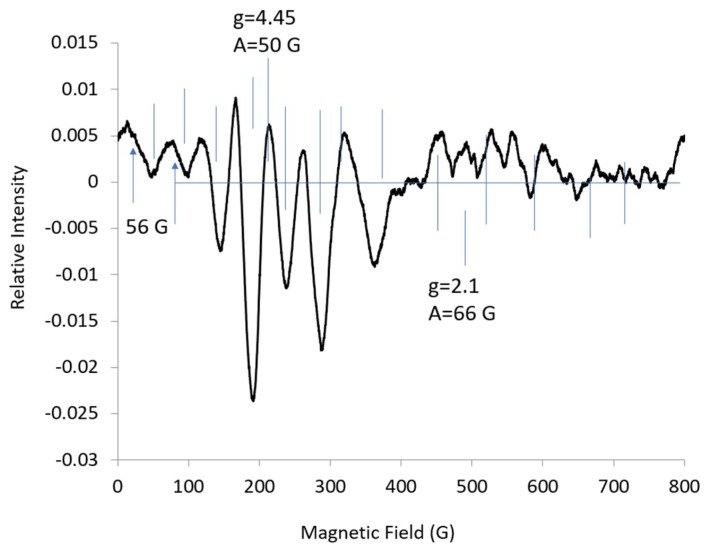
Second harmonic EPR spectrum at L-band (1.37 GHz) for CoEDTA taken from Figure 3, with Bessel function, 4%; temp., 18 K; microwave power, 8 dB; 25 scans averaged. The horizontal line is the baseline. The vertical lines designate ***g_eff_*** values and hyperfine lines. The two lines split by 56 G appear to be attributed to ***A***-max.

The EPR spectrum for CoEDTA at 18 K at L-band (1.37 GHz) was resolved into multiple lines (Figure 3). The intensity for the eight hyperfine lines centered at ***g_eff_*** = 7.83 for | ± 3/2> at L-band (18 K) was not enough to assign EPR parameters. The spectrum was attributed mainly to the | ± 1/2> state. The spacing between the lines and the line widths varied so that all the lines were not attributed to a single hyperfine pattern from a single ***g_eff_***-value. It was difficult to assign which lines were S-shaped and centered about ***g_eff_***-mid, and which lines were hill-shaped and centered about ***g_eff_***-max, partially because there was overlap at the lower microwave frequencies. A second harmonic (second derivative) spectrum was obtained to enhance the resolution of sharp lines over broader lines and to get a better look at the line shape (Figure 4). It also was difficult to assign the lines at ***g_eff_*** = 7.83 in the second harmonic spectrum due to overlap and the low intensity of the lines. The low intensity of the lines was consistent with the low intensity lines at S-band. Many of the remaining lines were attributed to Co hyperfine lines with an ***A***-value about 50 G (Figure 4). Much of the line shape for these lines was below the baseline, which indicates that the lines correspond to ***g_eff_***-mid.

### 2.3. Simulations with EPR Parameters for L-band Spectra

#### 2.3.1. Simulations for L-band Spectra with Parameters Varied to Access the Sensitivity of the Simulations

**Figure 5:**
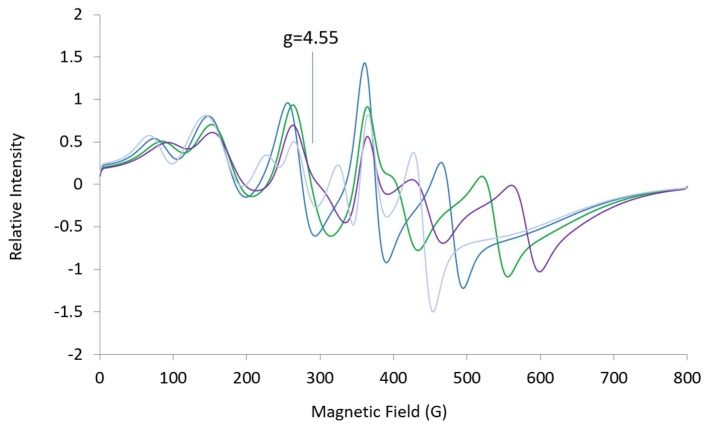
Simulated L-band (1.37 GHz) spectra: ***A*** = [369, 508, 100], ***g_eff_*** = [4.96, 4.55, 1.94], ***HStrain*** = [150, 150, 600] (blue); ***g***-mid = 4.04 (green), 3.75 (purple), 4.96 (light blue). Changes imply that two high-field lines are sensitive to ***g***-mid.

**Figure 6:**
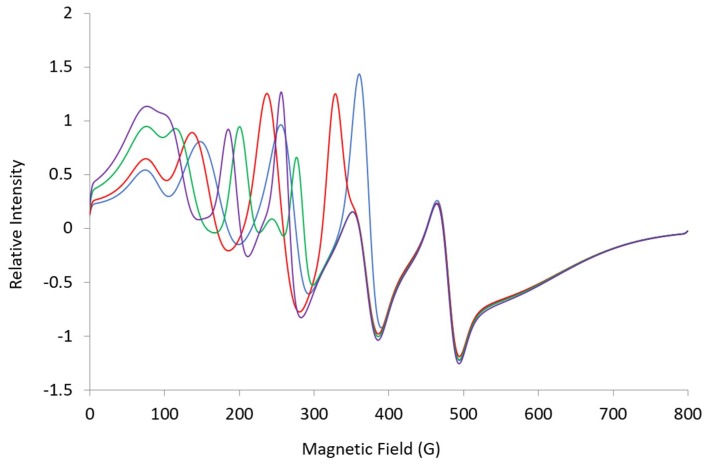
Simulated L-band (1.37 GHz) spectra: ***A*** = [369, 508, 100], ***g_eff_*** = [4.96, 4.55, 1.94], ***HStrain*** = [150, 150, 600] (blue); ***g***-max = 5.5 (red), 6.5 (green), 7.0 (purple). Changes show lines sensitive to ***g***-max.

**Figure 7:**
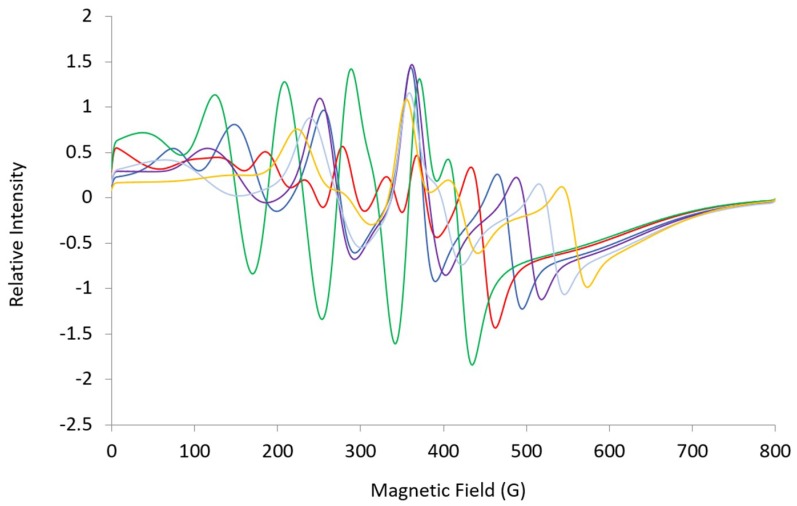
Simulated L-band (1.37 GHz) spectra: ***A*** = [369, 508, 100], ***g*** = [4.96, 4.55, 1.94], ***HStrain*** = [150, 150, 600] (blue); ***A***-mid = 450 MHz (red), 400 MHz (green), 550 MHz (purple), 600 MHz (light blue), 650 MHz (yellow). Changes imply that L-band simulations are sensitive to ***A***-mid.

**Figure 8:**
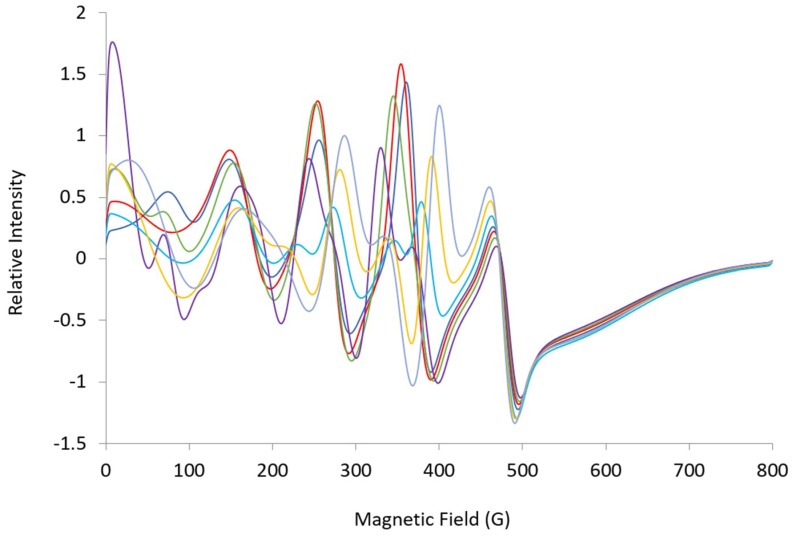
Simulated L-band (1.37 GHz) spectra: ***A*** = [369, 508, 100], ***g_eff_*** = [4.96, 4.55, 1.94], ***HStrain*** = [150, 150, 600] (blue); ***A***-low field = 350 MHz (red), 330 MHz (green), 300 MHz (purple), 400 MHz (light blue), 420 MHz (orange), 440 MHz (light purple). Changes in ***A***-low field affect lines throughout the L-band spectra expected for the high field line.

The positions of these lines varied with a change in input of EPR parameters, such as the ***g_eff_***-value or ***A***-value (Figure 5, Figure 6, Figure 7 and Figure 8, hypothetical EPR parameters), indicating that the EPR parameters were sensitive to simulated L-band spectra.

#### 2.3.2. Simulation of L-band Spectra for CoEDTA

**Figure 9:**
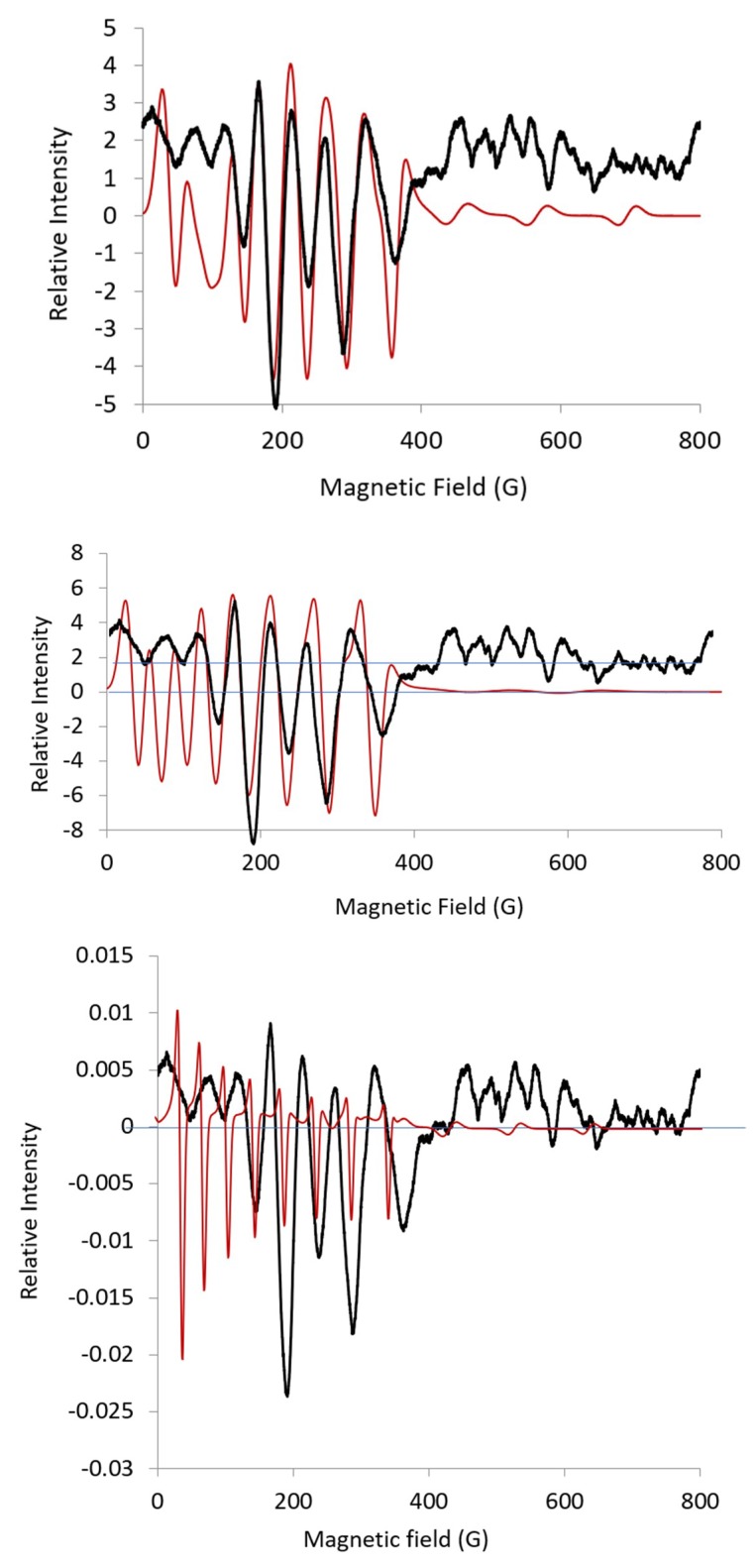
Second harmonic L-band spectrum (black) for CoEDTA as described in Figure 4. Simulation (red) using EPR parameters: ***g*** = [6.0, 5.0, 2.1], ***A*** = [200, 300, 162], ***HStrain*** = [500, 25, 50], bottom spectrum. Simulation (red) using EPR parameters: ***g*** = [5.0, 4.32, 2.10]; ***A*** = [244, 300, 162]; ***HStrain*** = [133, 140, 200]; middle spectrum spectrum; simulation (red) using EPR parameters ***g*** = [5.08, 4.14, 2.05]; ***A*** = [293, 221, 201]; ***HStrain*** = [174, 116, 81]; final simulation obtained running Monte Carlo option from EasySpin over a weekend with ***Vary.g*** = [0.2, 0.2, 0.2]; ***Vary.A*** = [50, 50, 50]; ***Vary.HStrain*** = [50, 50, 50]; center values: ***g*** = [5.0, 4.3, 2.0]; ***A*** = [300, 244, 162]; ***HStrain*** = [178, 123, 50].

In my opinion, a good procedure to fit the L-band spectrum for CoEDTA would involve using the second harmonic spectrum and limiting variation in the EPR parameters. The line shape in the experimental second harmonic spectrum was consistent with hyperfine lines about ***g_eff_***-mid. The hyperfine value, ***g_eff_***-mid, was taken from four sharp lines in the middle of the spectrum; this hyperfine value was not varied and ***g_eff_***-mid was not varied, as shown in the simulation in Figure 9, bottom simulated spectrum. The values for ***g_eff_***-max were arbitrarily taken as ***g_eff_***-max = 6.2 and ***A***-max = 200 MHz. The lines for ***g_eff_*** -max and ***A***-max were broadened out, ***HStrain*** = [500, 25, 50]. The center lines fit well as expected, but the intensity of the lines increased when moving from a higher field to a lower field. Next, the line widths were varied using ***HStrain*** as follows: start with ***HStrain*** = [900, 100, 100] and vary by ***HStrain*** = [0, 100, 100] (not shown). Although line widths can be broadened separately using terms involving ***g***, ***A***, or ***D***, all these broadening mechanisms were combined using the ***HStrain*** parameter, where modeling of the broadening with ***HStrain*** usually is “absolutely sufficient,” as specified in the EasySpin manual [13]. Again, it was noted that the hyperfine lines about ***g_eff_***-max were broadened out, resulting in little information about the parameters ***g_eff_***-max and ***A***-max. Next ***g_eff_***-max: 5.02 ± 2, ***A***-max: 214 ± 200; and ***HStrain***-max: 500 ±300, then ***HStrain*** = 200 ± 100 were varied, whereby the fitting routine changed the shape of the lines by moving ***g_eff_***-max to ***g_eff_***-mid: 4.32 and the lines were more S-shaped than cone-shaped (Figure 9, middle simulated spectrum). The parameters are ***g*** = [5.0, 4.32, 2.1], ***A*** = [244, 300, 162], and ***HStrain*** = [133, 140, 200]. ***A***-values of 38 G for ***A***-mid and 41 G for ***A***-max were obtained from the simulation. The ***HStrain*** parameter was set to show the contributions of ***g_eff_***-mid and ***A***-mid compared with ***g_eff_***-max and ***A***-max (Figure S1). Then, ***HStrain*** was varied to give the final simulation with resolved contributions from ***A***-max and ***A***-mid (Figure 9, top simulated spectrum). Some of the lines had shoulders, which accounted for the superposition of lines and resulted in unequal splittings between the lines. This accounted, in part, for a decrease in the intensity of the low-field lines. All the lines fit well to the experimental spectrum.

## 3. Discussion

CoEDTA may be a six-coordinate structure, but it could be a seven-coordinate structure, where a solvent molecule including water forms a complex like the complex formed for iron, i.e., FeEDTA-H_2_O, -carbonate, or −O_2_^2−^ [14]. The mixture of nitrogen and oxygen donor atoms mimics some of the biological sites.

The difference in the spacing between the lines in the EPR spectrum at L-band (Figure 3) can be explained by the rhombic g-values obtained from the L-band simulation; see Table 1. This was consistent with an E/D value of about 0.1, as obtained from the rhombogram [15]. Looking back at the first harmonic spectrum (Figure 3), the low-field lines marked by arrows have contributions from ***A***-max, but it took simulations of the second harmonic to assign these lines.

The value in determining an additional parameter, ***A***-mid, is that ***A***-mid is sensitive to the electron density in the | ± 1/2> orbital for the Co complex. The differences in line shape and EPR parameters for Co-doped Ba(Zn_1/3_Ta_2/3_)_O3_, CoBSA, CoCzcP, and Co(D4)(dca) [9,10,11,12] and CoEDTA suggested that low-frequency spectra were sensitive to differences in the electronic configuration. In summary, the best values appeared to be ***g*** = 7.8 and ***A*** = 84 G for the | ± 3/2> doublet and ***A***-min = 54 G for | ± 1/2> doublet from the X-band spectrum; and ***g***-max = 5.08, g-mid 4.14, ***A***-max = 41 G; ***A***-mid = 38 G from the simulation of the second harmonic L-band spectrum. Note that the g-values were the effective g-values.

Better resolution can be used to better determine small changes in the EPR parameters, for example, by titrating a single change of ligand or transfer of Co to a new binding site (Figure 5, Figure 6, Figure 7 and Figure 8). Changes were detected in the position of lines in the L-band spectrum (Figure 5, Figure 6, Figure 7 and Figure 8), but simulations of the second harmonic spectrum were necessary to obtain EPR parameters because the lines from A-max and A-mid were superimposed. In this example, we obtained lines from the experimental spectrum, assuming only lines for A-mid overestimated A-mid. The best value for A-mid was the value from the simulation of the second harmonic spectrum. More studies should follow that use a tetrahedral or five-coordinate configuration and low-spin instead of high-spin cobalt complexes.

## 4. Materials and Methods

### 4.1. Sample Preparation

CoEDTA (1 mM) samples (pink color) were made in 50 mM phophate (pH 7) and 20% glycerol.

### 4.2. EPR Spectrometers

Spectra were recently obtained from a low-frequency spectrometer, as described in [9]. In brief, the L-band and S-band bridges and the loop-gap resonators were homebuilt by the Dr. James S. Hyde laboratory at the National Biomedical EPR Center at the Medical College of Wisconsin (Milwaukee, WI, USA) and are located therein. The X-band (9.631 GHz) spectrometer at the National Biomedical EPR Center was a Bruker E500 ELEXSYS DM0101 cavity.

The second harmonic spectra (similar to the second derivative spectra) were obtained using an in-house program, SUMSPC, available at the National Biomedical EPR Center [16].

Simulations were completed using an online version of EasySpin [13]. Some of the simulations come with a warning that there are looping transitions and possible discontinuities at the ends of the spectrum [17,18].

## Figures and Tables

**Table 1:** Electron paramagnetic resonance (EPR) parameters for high-spin the Co complex of ethylene diamine tetraacetic acid (CoEDTA) from X-band and L-band spectra^a^.

EPR Parameter	*g*-_max_	*g*-_mid_	*g*-_max_	*A*-_max_	*A*-_mid_	*A*-_min_	*g*-_max_	*g*-_mid_	*g*-_max_	*A*-_max_	*A*-_mid_	*A*-_min_
High spin	| ± 3/2>						| ± 1/2>					
CoEDTA												
X-band (Exp)	7.8	---	---	84 G	---	---	~5	~4.4	2.1	---	---	54 G
L-sim	---	---	---	---	---	---	5.08	4.14	2.05	41 G	38 G	---
(Second harmonic)												

^a^ The g-values are the effective g-values.

## Supplementary Materials

Supplementary Materials can be found at https://www.mdpi.com/1422-0067/20/10/2385/s1.

## Abbreviations

Co	Cobalt
CoEDTA	Cobalt complex of ethylene diamine tetraacetic acid
EPR	Electron paramagnetic resonance
G	Guass
