# Supplementary material for: Resolved Hyperfine at L-band for High-Spin CoEDTA, A Model for Co Sites in Proteins
Source: Int J Mol Sci. 2019 May 14;20(10):2385. doi: 10.3390/ijms20102385 (PMC6566446; doi:10.3390/ijms20102385)
Supplement: Supplementary file 1 [file ijms-20-02385-s001.pdf]

International Journal of *Molecular Sciences*

MDPI

Article – Supplementary Materials

Resolved Hyperfine at L-band for High Spin CoEDTA, A Model for Co Sites in Proteins

William E. Antholine

Department of Biophysics, Medical College of Wisconsin, Milwaukee, WI 53226, USA; wantholi@mcw.edu; Tel.: 414-955-4032

Received: 16 April 2019; Accepted: 10 May 2019; Published: date

Supplemental Figure

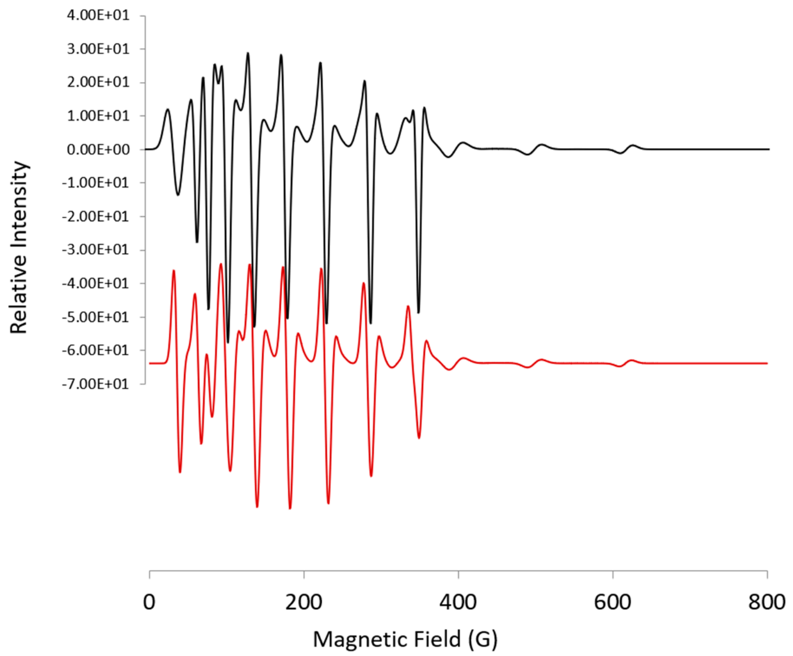

Figure S1. Simulated L-band spectra: ***g*** = [5, 4.31, 2.14]; ***A*** = [300, 24, 162]; mwFreq = 1.37 GHz; ***HStrain*** = [100, 25, 50] black trace, cone-shaped lines dominate; ***HStrain*** = [50, 50, 50] red trace, S-shaped lines dominate.

© 2019 by the authors. Submitted for possible open access publication under the terms and conditions of the Creative Commons Attribution (CC BY) license (http://creativecommons.org/licenses/by/4.0/).
